# Lingonberry Improves Hepatic Lipid Metabolism by Targeting Notch1 Signaling

**DOI:** 10.3390/antiox11030472

**Published:** 2022-02-27

**Authors:** Susara Madduma Hewage, Kathy K. W. Au-Yeung, Suvira Prashar, Charith U. B. Wijerathne, Karmin O, Yaw L. Siow

**Affiliations:** 1 Canadian Centre for Agri-Food Research in Health and Medicine, St. Boniface Hospital Research Centre, Winnipeg, MB R2H 2A6, Canada; smaddumahewage@sbrc.ca (S.M.H.); kauyeung@sbrc.ca (K.K.W.A.-Y.); suvira.prashar@agr.gc.ca (S.P.); cwijerathne@sbrc.ca (C.U.B.W.); 2 Department of Physiology & Pathophysiology, University of Manitoba, Winnipeg, MB R3E 0J9, Canada; 3 Department of Animal Science, University of Manitoba, Winnipeg, MB R3T 2N2, Canada; 4 Agriculture and Agri-Food Canada, St. Boniface Hospital Research Centre, Winnipeg, MB R2H 2A6, Canada

**Keywords:** nonalcoholic fatty liver disease (NAFLD), high-fat diet (HFD), lingonberry, notch signaling, lipid metabolism, palmitic acid

## Abstract

Impaired hepatic lipid metabolism is a hallmark of non-alcoholic fatty liver disease (NAFLD), which has no effective treatment option. Recently, Notch signaling has been identified as an important mediator of hepatic lipid metabolism. Lingonberry (*Vaccinium vitis-idaea* L.) is an anthocyanin-rich fruit with significant lipid-lowering properties. In this study, we examined how lingonberry influenced Notch signaling and fatty acid metabolism in a mouse model of NAFLD. Mice (C57BL/6J) fed a high-fat diet (HFD) for 12 weeks developed fatty liver and activated hepatic Notch1 signaling. Lingonberry supplementation inhibited hepatic Notch1 signaling and improved lipid profile by improving the expression of the genes involved in hepatic lipid metabolism. The results were verified using a palmitic-acid-challenged cell model. Similar to the animal data, palmitic acid impaired cellular lipid metabolism and induced Notch1 in HepG2 cells. Lingonberry extract or cyanidin-3-glucoside attenuated Notch1 signaling and decreased intracellular triglyceride accumulation. The inhibition of Notch in the hepatocytes attenuated sterol-regulatory-element-binding-transcription-factor-1 (*SREBP-1c)*-mediated lipogenesis and increased the expression of carnitine palmitoyltransferase-I-alpha (*CPTIα*) and acyl-CoA oxidase1 *(*ACOX1)*.* Taken together, lingonberry’s hepatoprotective effect is mediated by, in part, improving hepatic lipid metabolism via inhibiting Notch1 signaling in HFD-induced fatty liver.

## 1. Introduction

Nonalcoholic fatty liver disease (NAFLD) is the most common chronic liver disease in the world [1]. NAFLD is defined by accumulating lipids (mainly triglyceride) comprising more than 5% of the liver weight [2]. NAFLD comprises a broad pathological spectrum ranging from simple fatty liver (steatosis) to fatty liver with hepatic inflammation (steatohepatitis), fibrosis and cirrhosis [3]. The global prevalence of NAFLD has become nearly one-third of the population with the rapid growth rate of obesity and sedentary lifestyles [1]. Elevated hepatic lipid influx, impaired lipid metabolism and decreased lipid export from the liver are the primary causes of fatty liver [3]. Chronic lipid accumulation in the liver triggers oxidative stress and inflammation, the driving forces of progressing steatosis to nonalcoholic steatohepatitis (NASH). Impaired hepatic lipid metabolism is common in NAFLD patients and in high-fat-diet (HFD)-fed rodent models [4]. It has been demonstrated that hepatic de novo lipogenesis is three-fold higher in human NAFLD subjects than in control subjects [5]. Furthermore, elevated fatty acid synthesis attenuates lipolysis/fatty acid oxidation by inhibiting fatty acid transportation into the mitochondrial matrix [6]. Therefore, hepatic lipid metabolism has been identified as a potential therapeutic target to improve NAFLD [7].

Notch is an intracellular signaling mechanism that transduces signals to the nucleus following the activation of its transmembrane cell-surface receptors [8]. Notch signaling plays a crucial role in the development, repair and homeostasis of the liver [9]. However, dysregulation of Notch signaling in the liver is associated with impaired lipid metabolism, inflammation and fibrosis [10]. Recent studies indicate that the Notch signaling pathway is positively correlated with the fatty liver [11]. Notch downstream signaling is activated by intracellular cleavage of the Notch receptor. Upon activation, the cleaved Notch receptor or the Notch intracellular domain (NICD) acts as the downstream signal transducer of the Notch pathway [12]. Among the four Notch receptors, Notch receptor 1 (Notch1) expression is elevated in NAFLD, as evidenced by the liver biopsy samples obtained from NAFLD patients and the liver tissue of HFD-fed mice [13]. Increased hepatic expression of Notch1 promotes insulin resistance and gluconeogenesis in HFD-fed mice [14]. Further, a significant elevation in Notch1 expression has been observed in mice fed a NASH diet [15]. Another study has demonstrated that inhibition of Notch signaling attenuated carbon-tetrachloride (CCl_4_)-induced liver fibrosis in rats, proposing the hepatoprotective role of pharmacological inhibition of Notch in liver damage [16]. Therefore, it has been suggested that the Notch signaling pathway plays a role in the development and progression of NAFLD by interfering with multiple steps in lipid metabolism.

Although the prevalence of NAFLD has increased globally, there are limited treatment options [2]. Since improving hepatic lipid metabolism may stop the progression of NAFLD, there has been a continuous search for potential disease management solutions. These include lifestyle and dietary interventions. Lingonberry (*Vaccinium vitis-idaea* L.) is an evergreen dwarf woody plant that produces small reddish berries rich in anthocyanins [17]. Lingonberry has shown promising health benefits, including antioxidant, anti-inflammatory and lipid-lowering activity in in vitro and in vivo models [18,19,20,21,22,23]. Previously, we reported that lingonberry anthocyanins protect cardiac cells from oxidative-stress-induced apoptosis via suppressing caspase-3 activation [22]. Lingonberry also protects mice from HFD-induced chronic kidney disease by inhibiting the nuclear factor kappa-light-chain-enhancer of activated b cell (NF-κB)-mediated inflammation [23]. We have further observed that lingonberry supplementation improves liver function [24]. Additionally, consumption of lingonberry prevents adipocyte hypertrophy and protects vascular endothelial dysfunction, inhibiting oxidative stress and inflammation in HFD-fed mice [25]. The antioxidant properties of lingonberry have been well-established [21,22,24,25], and the potential crosstalk of Notch1 signaling with the antioxidant pathway has been demonstrated in the heart [26]. However, the effect of lingonberry on Notch signaling and the underlying mechanism for the lipid-lowering properties of lingonberry are not fully understood. Therefore, the current study aimed to investigate the impact of lingonberry supplementation on Notch-mediated lipid metabolism using a mouse model of NAFLD induced by HFD feeding.

## 2. Materials and Methods

### 2.1. Animal Model

Male C57BL/6J mice were purchased from Central Animal Care Services (University of Manitoba, Winnipeg, MB, Canada) and were housed two per cage in a temperature- and humidity-controlled room with a 12 h dark–12 h light cycle. At the age of six weeks, the animals were divided into three groups. Each group was given a (1) control diet (D12450J, Research Diets Inc., Brunswick, NJ, USA) containing 11% kcal fat, 18% kcal protein and 71% kcal carbohydrate, or (2) an HFD (D12492) containing 62% kcal fat, 18% kcal protein and 20% kcal carbohydrate, or (3) an HFD supplemented with (5% *w*/*w*) Manitoba wild lingonberry (D17022206). The fat sources of HFD were derived from 90% lard and 10% soybean oil. The three diets listed above were given ad libitum for 12 weeks. The average feed intake and weight gain of the animals were recorded throughout the trial. The animals were sacrificed at the end of the feeding period, and the liver tissues were collected. All the procedures were performed in accordance with the Guide to the Care and Use of Experimental Animals published by the Canadian Council on Animal Care and approved by the University of Manitoba Protocol Management and Review Committee.

### 2.2. Cell Culture

Human hepatoma cells (HepG2, cell line: HB-8065) were purchased from American Type Culture Collection (Manassas, VA, USA). HepG2 cells were cultured in Dulbecco’s modified eagle medium (DMEM) (VWR, Radnor, PA, USA) supplemented with 10% fetal bovine serum (Hyclone Laboratories Inc., Logan, UT, USA) at 37 °C in a humidified atmosphere containing 5% CO_2_. The cells were subcultured before they reached 90% confluency, and all the experiments were performed using the cells in between 5–20 passages. The cells were seeded in 6-well plates or 60 mm dishes at a 1 × 10^5^ cells/mL density and incubated for 24 h. The cells were pretreated with lingonberry extract, cyanidin-3-glucoside (C3Glu) (Cerilliant Corp., Round Rock, TX, USA) and γ-secretase inhibitor N-[N-(3,5-difluorophenacetyl-L-alanyl)]-(S)-phenylglycine t-butyl ester (DAPT, Abcam, Cambridge, UK) alone or DAPT plus lingonberry extract for 30 min followed by incubation with palmitic acid dissolved in 10% bovine albumin serum (Sigma-Aldrich. St. Louis, MO, USA) for another 24 or 48 h.

### 2.3. Triglyceride and Total Cholesterol Assays

Hepatic lipids were extracted from the liver tissues using the Folch method [27,28]. Briefly, the tissues were homogenized with a solution mixture containing chloroform, methanol and distilled water (*v*:*v*:*v* ratio of 4:2:3). The homogenate was centrifuged to recover the liquid phase, and the lipids, separated into the chloroform phase, were dried using nitrogen gas. The dried lipids were then resuspended in ethanol. For intracellular triglycerides, the cells were collected and resuspended in PBS, followed by sonication. Triglycerides and total cholesterol in the extracts were measured using commercial kits according to the manufacturer’s instructions (Sekisui Diagnostics, Burlington, MA, USA) [29].

### 2.4. Western Immunoblotting

The protein levels of Notch1 and cleaved Notch1/Notch1 intracellular domain (NICD1) were detected using western immunoblotting. Total proteins were extracted from mouse liver tissues in a protein lysis buffer containing 20 mM Tris at pH 7.4, 150 mM NaCl, 1 mM EGTA, 1 mM EDTA, 2.5 mM sodium pyrophosphate, 1 mM β-glycerophosphate, 1 mM sodium orthovanadate, 2.1 μM leupeptin, 1 mM PMSF and 1% (*v*/*v*) Triton X-100. Extracted proteins were quantified and separated in a 10% SDS polyacrylamide gel as previously described [23,24]. Following electrophoresis and electrotransfer onto nitrocellulose membrane, the blots were probed with rabbit anti-Notch1 monoclonal antibody or rabbit anti-NICD1 monoclonal antibody (Cell Signaling Technology, Danvers, MA, USA). The membranes were then reprobed with rabbit anti-β-actin monoclonal antibody (Cell Signaling Technology) to ensure equal loading of the samples. All the blots were incubated with HRP-conjugated anti-rabbit IgG secondary antibodies (Cell Signaling Technology). Proteins were visualized by using an ECL detection system (Bio-Rad, Hercules, CA, USA) and quantified using Quantity One software version 4.6.8 for Windows (Bio-Rad).

### 2.5. Real-Time qPCR

Relative mRNA expression of *Notch1,* hairy and enhancer of split-1 (*HES1*), fatty acid translocase *(CD36),* diacylglycerol acyltransferase 1 and 2 (*DGAT1* and *DGAT2*), acyl-CoA oxidase1 *(ACOX1),* carnitine palmitoyltransferase-I-alpha *(CPTIα),* acetyl-CoA carboxylase 1 *(ACC1)* and sterol regulatory element-binding transcription factor-1 *(SREBP-1c)* were measured using the real-time qPCR technique. Briefly, total RNA was extracted, and cDNA was constructed from the HepG2 cells and mouse liver tissues as previously described [23,30]. The qPCR mixture was prepared by mixing 100 ng of cDNA, 1X iTaq Universal SYBR Green Supermix (Bio-Rad), 300 nM per primer and RNase-free water in a total reaction mixture of 20 μL. The qPCR was performed using a previously described protocol [23]. The data were analyzed using the comparative CT method with gene expression levels normalized to that of the housekeeping gene β-Actin [31]. Primer sequences used for the RT-qPCR are shown in Table 1.

### 2.6. Histological Staining

A portion of the liver tissue was fixed in 10% neutral-buffered formalin and embedded in paraffin, then sectioned into a thickness of 5 μm [32]. The paraffin sections were stained with hematoxylin and eosin (H&E) to evaluate the morphological changes in the liver. The cells were seeded at a density of 4 × 10^4^ cells/chamber in a chamber slide (Thermo Fisher Scientific, Waltham, MA, USA) and incubated for 24 h. The cells were pretreated with lingonberry extract, C3Glu and DAPT or DAPT plus lingonberry extract for 30 min and incubated with palmitic acid for another 48 h. The cells were stained with Oil Red O to visualize neutral lipid accumulated in the cells [24]. Briefly, the cells were fixed in 10% formal calcium and immersed in 100% propylene glycol and stained with 0.7% Oil Red O solution followed by immersion in 85% propylene glycol. After rinsing with distilled water, the cells were counterstained with Mayer’s Hematoxylin. The images were taken using an Olympus BX43 Upright Light Microscope (Olympus Corp., Tokyo, Japan) equipped with a Q-color 3 digital camera and analyzed using Image-Pro Plus 7.0 (Media Cybernetics, Rockville, MD, USA).

### 2.7. Statistical Analysis

Results were analyzed using one-way ANOVA followed by Newman–Keuls multiple comparisons test and expressed as mean ± standard deviation (SD). ProStat Version 6 software (Poly Software International, Pearl River, NY, USA) was used to perform all the statistical analyses. A *p*-value of less than 0.05 was considered statistically significant.

## 3. Results

### 3.1. Lingonberry Supplementation Inhibits Hepatic Notch1 Signaling

**Figure 1:**
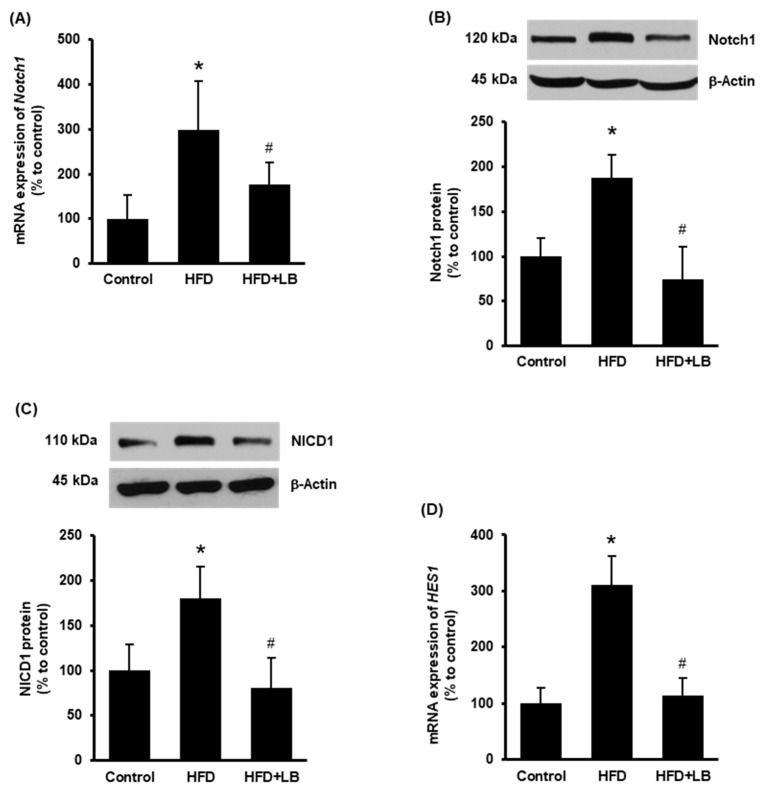
Effect of HFD feeding and lingonberry supplementation on hepatic Notch1 activation. Mice were fed a control diet, HFD or HFD supplemented with 5% (*w*/*w*) Manitoba wild lingonberry powder for 12 weeks. Liver Notch1 (**A**) relative mRNA and (**B**) protein expression were measured. (**C**) Protein expression of the NICD1 was measured in the liver. (**D**) Hepatic relative mRNA expression of *HES1* was measured. The real-time qPCR technique was used to measure relative mRNA expressions. The results are expressed as mean ± SD (*n* = 4 to 6). * *p* < 0.05 when compared with the value obtained from the control group. ^#^ *p* < 0.05 when compared with the value obtained from the HFD group.

Increased expression of hepatic Notch1 receptor is observed in NAFLD in human subjects and mouse models [13]. Therefore, Notch1 expression was first examined in the mouse liver tissues. HFD feeding for 12 weeks increased both mRNA and protein levels of Notch1 (Figure 1A,B). Supplementation of 5% (*w*/*w*) Manitoba lingonberry with HFD significantly reduced Notch1 (Figure 1A,B) expression in the liver. Next, the protein level of cleaved Notch1 (NICD1) and mRNA expression of *HES1* were studied to confirm Notch1 activation. HFD feeding increased liver NICD1 protein and *HES1* mRNA levels (Figure 1C,D), while lingonberry supplementation significantly decreased these changes (Figure 1C,D).

### 3.2. Lingonberry Supplementation Attenuates Liver Lipid Accumulation

**Figure 2:**
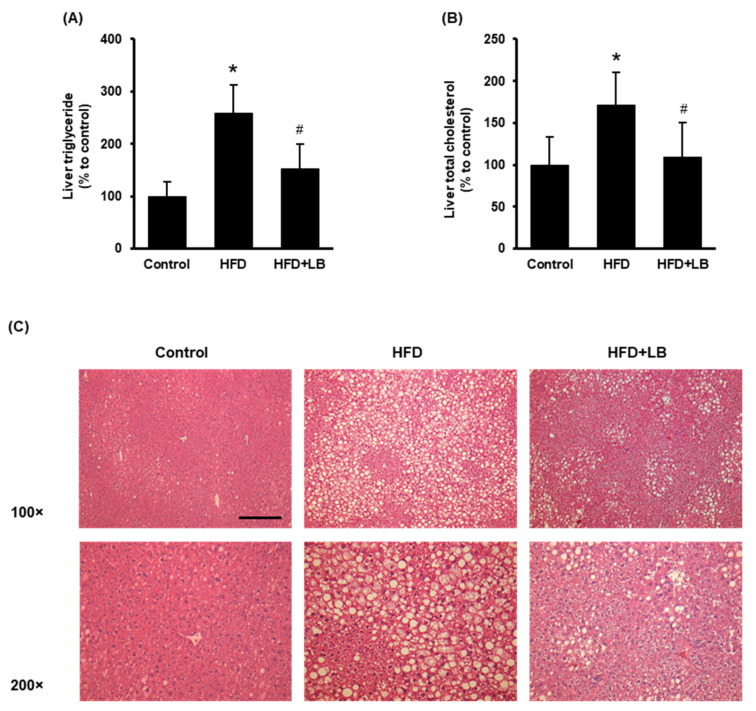
Effect of HFD feeding and lingonberry supplementation on liver lipid accumulation. Mice were fed a control diet, HFD or HFD supplemented with 5% (*w*/*w*) Manitoba wild lingonberry powder for 12 weeks. Liver (**A**) triglyceride and (**B**) total cholesterol levels were measured. (**C**) Paraffin sections of the liver tissues were stained with hematoxylin and eosin (H&E) to examine the histological changes (Scale bar = 100 μm, magnifications 100× and 200×). The results are expressed as mean ± SD (*n* = 5 to 6). * *p* < 0.05 when compared with the value obtained from the control group. ^#^ *p* < 0.05 when compared with the value obtained from the HFD group.

HFD feeding increased liver lipid accumulation identified by elevated hepatic triglyceride and total cholesterol levels (Figure 2A,B). Lingonberry supplementation significantly reduced hepatic triglyceride and total cholesterol accumulation (Figure 2A,B). The H&E staining revealed noticeable accumulation of lipid droplets identified by larger vacuoles in the liver tissue sections of HFD-fed mice compared to those fed a control diet (Figure 2C). A smaller and reduced number of vacuoles were observed in the liver sections of mice provided an HFD supplemented with lingonberry (Figure 2C). Throughout the experiment, the animals did not show any significant change in feed (diet) intake. At the end of the 12-week feeding period, HFD-fed mice showed a significant weight gain compared to the control mice. However, lingonberry supplementation did not influence the body weight gain by HFD feeding. These data were reported in our previous study [24].

### 3.3. Lingonberry Supplementation Improves Liver Lipid Metabolism

**Figure 3:**
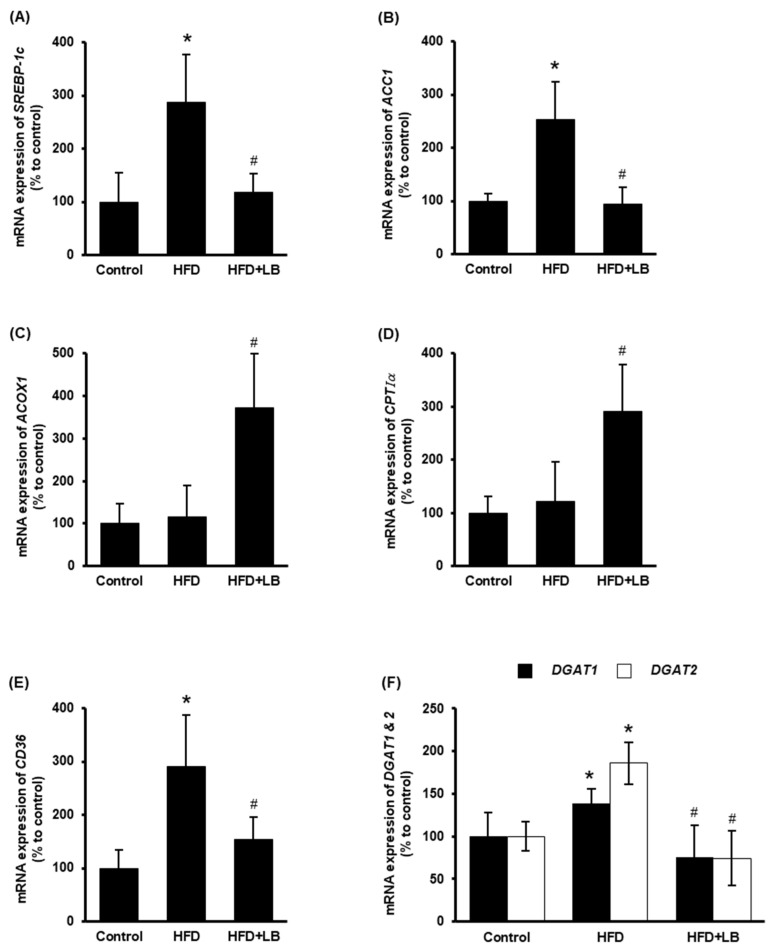
Effect of HFD feeding and lingonberry supplementation on liver lipid metabolism. Mice were fed a control diet, HFD or HFD supplemented with 5% (*w*/*w*) Manitoba wild lingonberry powder for 12 weeks. Relative mRNA expression of (**A**) *SREBP-1c*, (**B**) *ACC1*, (**C**) *ACOX1*, (**D**) *CPTIα*, (**E**) *CD36* and (**F**) *DGAT1* and *DGAT2* were measured in the liver using real-time qPCR. The results are expressed as mean ± SD (*n* = 6). * *p* < 0.05 when compared with the value obtained from the control group. ^#^ *p* < 0.05 when compared with the value obtained from the HFD group.

The impact of lingonberry on hepatic lipid metabolism was examined. *ACC1* is the rate-limiting enzyme for fatty acid biosynthesis, which is transcriptionally regulated by the transcriptional factor *SREBP-1c* [33]. HFD feeding increased the hepatic expression of *SREBP-1c* and *ACC1* (Figure 3A,B). Dietary supplementation of lingonberry significantly reduced the expression of both genes (Figure 3A,B). CPTIα regulates the rate-limiting step of mitochondrial fatty acid oxidation, while ACOX1 catalyzes the rate-limiting reaction of peroxisomal fatty acid oxidation [34,35]. Lingonberry markedly increased the hepatic mRNA levels of *ACOX1* and *CPTIα* (Figure 3C,D); however, no significant change was observed in mice fed an HFD (Figure 3C,D). Increased hepatic expression of *CD36* elevates hepatocyte lipid uptake and the progression of the fatty liver [36]. Moreover, DGAT1 and DGAT2 are the key enzymes that catalyze the formation of triglycerides from diacylglycerol and acyl-CoA [37]. Hepatic gene expression of *CD36*, *DGAT1* and *DGAT2* were markedly increased in mice fed an HFD (Figure 3E,F). Lingonberry supplementation reduced HFD-induced elevation in these gene expressions (Figure 3E,F).

### 3.4. Lingonberry Extract and C3Glu Reduce Intracellular Lipid Accumulation and Suppress Notch1 and SREBP-1c Signaling

**Figure 4:**
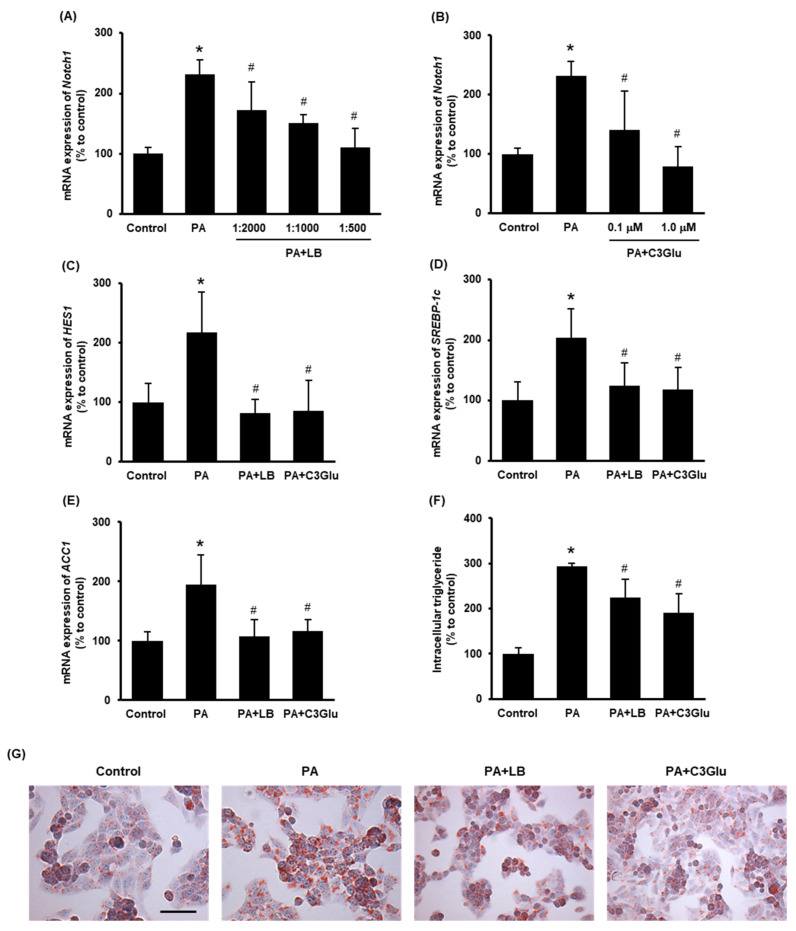
Effect of lingonberry extract and C3Glu on Notch signaling and lipogenesis in the HepG2 cells. Relative mRNA expression of *Notch1* was measured in the cells pretreated with different dilutions of (**A**) lingonberry extract (dilutions: 1:2000, 1:1000 or 1:500) or (**B**) various concentrations of C3Glu (0.1 or 1.0 μM) for 30 min and incubated with 0.3 mM palmitic acid for another 24 h. After selecting the optimum doses, the cells were pretreated with lingonberry extract (dilution 1:500) or C3Glu (1.0 μM) for 30 min and incubated with 0.3 mM palmitic acid for 24 h. Relative mRNA expression of (**C**) *HES1*, (**D**) *SREBP-1c* and (**E**) *ACC1* were measured using real-time qPCR. Separate sets of cells were treated with the same compounds as above but incubated with palmitic acid for 48 h. One set of cells was used to measure (**F**) intracellular triglyceride level, and the other set was used to stain with (**G**) Oil Red O to visualize intracellular lipids (Scale bar = 100 μm, magnification = 400×). The results are expressed as mean ± SD (*n* = 6). * *p* < 0.05 when compared with the value obtained from the control group. ^#^ *p* < 0.05 when compared with the value obtained from the palmitic-acid-treated group.

To further study the effect of lingonberry on Notch1 signaling and lipid metabolism, hepatocytes were challenged with palmitic acid. In preliminary experiments, we observed that treatment with 0.3 mM palmitic acid for 24 h recorded the maximum induction of *Notch1* gene expression (data not shown). The effect of lingonberry extract and C3Glu (one of the three anthocyanins found in lingonberry) on Notch signaling and induction of genes related to lipid synthesis was examined. Pretreatment of lingonberry extract (dilutions 1:2000, 1:1000 and 1:500) or C3Glu (concentrations 0.1 and 1.0 μM) significantly reduced palmitic-acid-induced *Notch1* mRNA expression (Figure 4A, B). Based on these results, 1:500 dilution of lingonberry extract and 1.0 μM C3Glu were selected to treat the cells for further experiments. Pretreatment of cells with lingonberry extract or C3Glu for 30 min significantly lowered palmitic-acid-induced *HES1*, *SREBP-1c* and *ACC1* mRNA expression (Figure 4C–E). To further confirm the effect of lingonberry extract and C3Glu on cellular lipid metabolism, intracellular triglyceride levels were measured in cells treated with palmitic acid for 48 h. Both lingonberry extract and C3Glu reduced palmitic-acid-induced intracellular triglyceride accumulation (Figure 4F). Additionally, Oil Red O staining showed increased cellular lipid droplets in the group treated with palmitic acid alone compared to the control group (Figure 4G). The cells pretreated with lingonberry extract or C3Glu exhibited fewer cellular lipid droplets compared to the cells treated with palmitic acid alone (Figure 4G).

### 3.5. Inhibition of Notch Signaling Improves Cellular Lipid Metabolism

**Figure 5:**
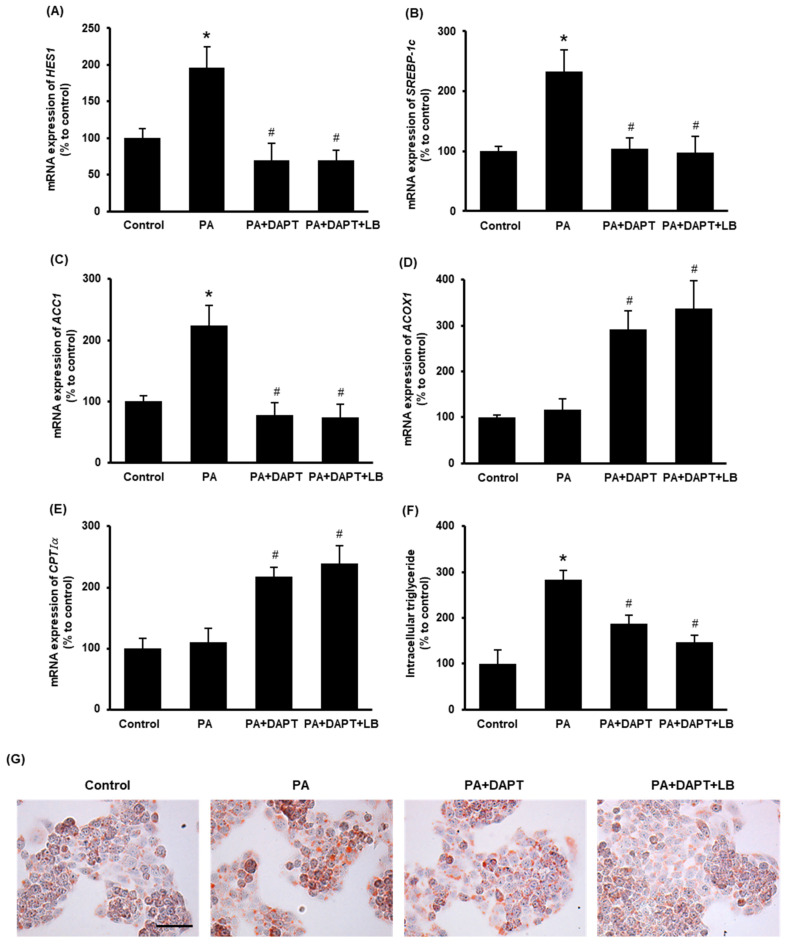
Effect of inhibition of the Notch signaling on lipid metabolism in the HepG2 cells. The cells were pretreated with vehicle or a γ-secretase inhibitor DAPT (10 μM) or DAPT plus lingonberry extract (dilution 1:500) for 30 min followed by incubation with palmitic acid (0.3 mM) for another 24 h. Relative mRNA expression of (**A**) *HES1*, (**B**) *SREBP-1c*, (**C**) *ACC1*, (**D**) *ACOX1* and (**E**) *CPTIα* was measured using real-time qPCR. Separate sets of cells were treated with DAPT as above but incubated with palmitic acid for 48 h. One set of cells was used to measure (**F**) intracellular triglyceride level, and the other set was stained with (**G**) Oil Red O to visualize intracellular lipids (Scale bar = 100 μm, magnification = 400×). The results are expressed as mean ± SD (*n* = 6). * *p* < 0.05 when compared with the value obtained from the control group. ^#^ *p* < 0.05 when compared with the value obtained from the palmitic-acid-treated group.

To inhibit Notch signaling, a γ-secretase inhibitor DAPT was used. Pretreatment of cells with DAPT (10 μM) or with DAPT plus lingonberry extract (dilution 1:500) inhibited palmitic-acid-induced mRNA expression of *HES1* (Figure 5A), a downstream target gene of Notch signaling. Incubation of the cells with DAPT or DAPT plus lingonberry extract significantly decreased the *SREBP-1c* and *ACC1* mRNA levels induced by palmitic acid (Figure 5B,C). Additionally, DAPT or DAPT plus lingonberry extract increased mRNA levels of *ACOX1* and *CPTIα* in palmitic-acid-treated hepatocytes (Figure 5D,E). In line with the results observed in the mouse liver tissues, cells incubated with palmitic acid alone did not significantly change the mRNA level of *ACOX1* and *CPTIα* (Figure 5D,E). There was no significant difference in gene expression in cells treated with DAPT or DAPT plus lingonberry extract. Next, intracellular triglyceride content was examined. Incubation of cells with palmitic acid for 48 h resulted in a significant increase in cellular triglyceride compared to the control cells (Figure 5F). Pretreatment of cells with DAPT or DAPT plus lingonberry extract markedly reduced palmitic-acid-induced intracellular triglyceride levels (Figure 5F). Another set of cells was stained with Oil Red O to visualize cellular lipid accumulation. Incubation of cells with palmitic acid for 48 h resulted in increased cellular lipid droplets compared to the control group (Figure 5G). The cells pretreated with DAPT or a combination of DAPT and lingonberry extract exhibited fewer cellular lipid droplets compared to the cells treated with palmitic acid alone (Figure 5G).

## 4. Discussion

The current study investigated the impact of lingonberry supplementation on hepatic Notch1 signaling and lipid metabolism using mice and hepatocytes. Our data indicate that supplementation with 5% (*w*/*w*) Manitoba wild lingonberry improved HFD-induced fatty liver by attenuating hepatic lipid accumulation. This was mediated, in part, through the inhibition of hepatic lipogenesis and the stimulation of fatty acid oxidation by suppressing Notch1 signaling in the liver. The favorable lipid-lowering effect of lingonberry was further supported by the in vitro results in hepatocytes treated with lingonberry extract or its anthocyanin (C3Glu).

Elevated Notch1 signaling was shown to stimulate liver lipid accumulation by inducing hepatic de novo lipogenesis in NAFLD by upregulating transcriptional activation of fatty acid synthesis genes [38]. The results from the current study are in line with those from previous studies that indicate HFD feeding results in activation of Notch1 signaling and lipid accumulation in the liver [13,39]. Lingonberry supplementation attenuated hepatic Notch1 expression and fatty liver in mice fed an HFD. To further understand the mechanism of lingonberry’s effect on Notch-mediated lipid metabolism, an in vitro hepatocyte model (HepG2) was used. Cells were incubated with palmitic acid, the most common saturated fatty acid found in the HFD. Similar to the results observed in HFD-fed mice, incubation of cells with palmitic acid increased cellular lipid accumulation, lipogenesis and Notch1 signaling. Pretreatment of cells with lingonberry extract inhibited Notch1 signaling, lipogenesis and cellular lipid accumulation induced by palmitic acid. Lingonberry is an anthocyanin-rich berry, with particular abundancies of cyanidin-3-galactoside (C3Gal), cyanidin-3-arabinoside (C3Ara) and cyanidin-3-glucoside (C3Glu) [21]. Anthocyanins are a group of water-soluble polyphenols that have been shown to have beneficial health effects against oxidative stress, inflammation and obesity [40]. Pretreatment of cells with C3Glu inhibited cellular lipogenesis and Notch1 signaling in the cells treated with palmitic acid. However, the other two anthocyanins, C3Gal and C3Ara, did not significantly change the Notch1 expression in palmitic-acid-treated cells (data not shown).

During Notch activation, the enzyme γ-secretase intracellularly cleaves the Notch receptor to yield NICD, the second messenger of the Notch pathway [8]. To confirm the involvement of Notch signaling in lingonberry’s lipid-lowering effect, a γ-secretase inhibitor, DAPT, was used [41]. Notch inhibition significantly lowered cellular lipid accumulation by ameliorating SREBP-1c-mediated lipogenesis and augmenting fatty acid oxidation. Similar findings were observed in HFD-fed mice with liver-specific RBPJ knockout (L-RBPJ) [38]; recombination-signal-binding protein for immunoglobulin kappa J region (RBPJ) is a DNA-binding protein that is essential for NICD to interact with its target DNA in the nucleus [12]. Another study reported that Notch1 deficiency increased fatty acid oxidation in the liver by elevating the expression of fatty acid oxidation genes [39]. In accordance with these findings, our results suggest that lingonberry extract or C3Glu reduced cellular lipid accumulation by decreasing lipogenesis and increasing fatty acid oxidation, in part through inhibiting Notch1 signaling. Further, elevated fatty acid oxidation might be a combined effect of Notch1 inhibition and involvement of other possible signaling pathways affected by lingonberry. Sirtuins (SIRTs 1–7) are a family of nicotinamide-adenine-dinucleotide (NAD)-dependent histone deacetylases that can regulate a variety of cellular processes, such as cellular energy metabolism and cell survival [42]. It has also been reported that SIRT4 levels are significantly reduced in obese patients with NAFLD [43]. Recent studies showed that SIRTs may play key roles in regulating insulin sensitivity/resistance and fatty acid β-oxidation [42,44,45]. Therefore, the effects of lingonberry on SIRT expression and insulin resistance in NAFLD warrant further research.

Increased hepatic de novo lipogenesis is a characteristic of NAFLD [5]. The formation of malonyl-CoA is the rate-limiting step of fatty acid biosynthesis, which is catalyzed by the enzyme ACC1 through the irreversible carboxylation of acetyl-CoA to form malonyl-CoA [46]. Increased hepatic expression of ACC1 is an indicator for elevated lipogenesis in the liver [47]. SREBP-1c promotes the expression of a family of genes involved in glucose utilization and fatty acid synthesis, including *ACC1*. Therefore, elevated hepatic SREBP-1c expression plays a major role in fatty liver by stimulating de novo lipogenesis [33]. In the current study, lingonberry supplementation attenuated lipogenesis by inhibiting the expression of *SREBP-1c* and *ACC1* in the liver of HFD-fed mice. The rate of fatty acid conversion into triglycerides is increased in NAFLD due to the continuous uptake and de novo synthesis of free fatty acids [48]. The enzymes DGAT1 and 2 catalyze the final step in triglyceride synthesis [37]. Although DGAT (1 and 2) catalyze the same reaction, they are not redundant in their respective functions. Overexpression of *DGAT1* is associated with higher triglyceride levels that are packed into very-low-density lipoprotein (VLDL) particles. In contrast, DGAT2 is more related to the synthesis of triglycerides that are stored in the cytosol. Deletion or knock-out of hepatic *DGAT* (*1* and *2*) has shown improved fatty liver in HFD-fed mouse models [49]. Therefore, together with SREBP-1c-mediated fatty acid synthesis, the DGAT (1 and 2) enzymes also contribute to the development of the fatty liver. In the current study, lingonberry supplementation decreased the expression of *DGAT1* and *DGAT2*, which might contribute to a low triglyceride content in the liver of mice fed an HFD. Furthermore, lingonberry supplementation inhibited the expression of *CD36*, a scavenger receptor for fatty acid uptake, which might lead to reduced hepatic fatty acid uptake in mice fed an HFD. Our findings are in line with those of Kowalska et al., who reported that lingonberry extract downregulates the gene expression of *DGAT1* and other genes in the mouse adipocytes [25]. Additionally, Ryyti et al. observed that supplementation of HFD with dry Finnish lingonberry powder significantly reduced hepatic mRNA expression of monoacylglycerol O-acyltransferase 1 (*MGAT1*), the enzyme that synthesizes diacylglycerols from monoacylglycerol [50].

Free fatty acids present in the hepatocytes can also be oxidized for energy generation [51]. Recent studies have shown that stimulation of hepatic fatty acid β-oxidation can function as a potential therapeutic approach to manage NAFLD [7]. The two most important steps of fatty acid β-oxidation are the transportation of cytosolic fatty acids into the mitochondrial matrix and desaturation of acyl-coenzyme A [51]. The mitochondrial matrix houses fatty acid β-oxidation, and therefore, cytosolic free fatty acids need to be transported across the two mitochondrial membranes to start their combustion. However, the mitochondrial membranes are impermeable for most fatty acids; this process is mediated through the carnitine palmitoyl shuttle [34]. CPTIα is the gatekeeper and the rate-limiting enzyme of the mitochondrial fatty-acid-transporting shuttle [34]. At elevated concentrations, malonyl-CoA reduces fatty acid β-oxidation by inhibiting the carnitine palmitoyl shuttle [52]. Therefore, increased lipogenesis indirectly suppresses fatty acid oxidation in the liver. On the other hand, *ACOX1* plays a significant role in fatty acid oxidation by initiating the rate-limiting reaction of peroxisomal fatty acid β-oxidation [35]. Although HFD feeding did not change the expression of *ACOX1* and *CPTIα*, lingonberry supplementation significantly increased the expression of these two genes in the liver. These results suggest that dietary supplementation of lingonberry attenuated HFD-induced hepatic lipid accumulation by inhibiting de novo lipid synthesis and improving fatty acid oxidation in the liver. However, the lipid-lowering effect of lingonberry was independent of body weight change.

The strengths and weaknesses of the current study should be considered. To the best of our knowledge, this is the first study to suggest that lingonberry supplementation can improve hepatic fatty acid synthesis and oxidation via inhibition of hepatic Notch1 signaling. Further, we identified that the anthocyanin C3Glu (found in lingonberry) could be one of the potential bioactive compounds responsible for the observed hepatoprotective effects against HFD feeding. However, future studies are warranted to determine the impact of other bioactive compounds that may contribute to such beneficial effects. Although we have shown Notch inhibition caused a significant elevation in fatty acid oxidation gene expression, the upstream mediators of Notch and fatty acid oxidation pathways have yet to be studied.

## 5. Conclusions

**Figure 6:**
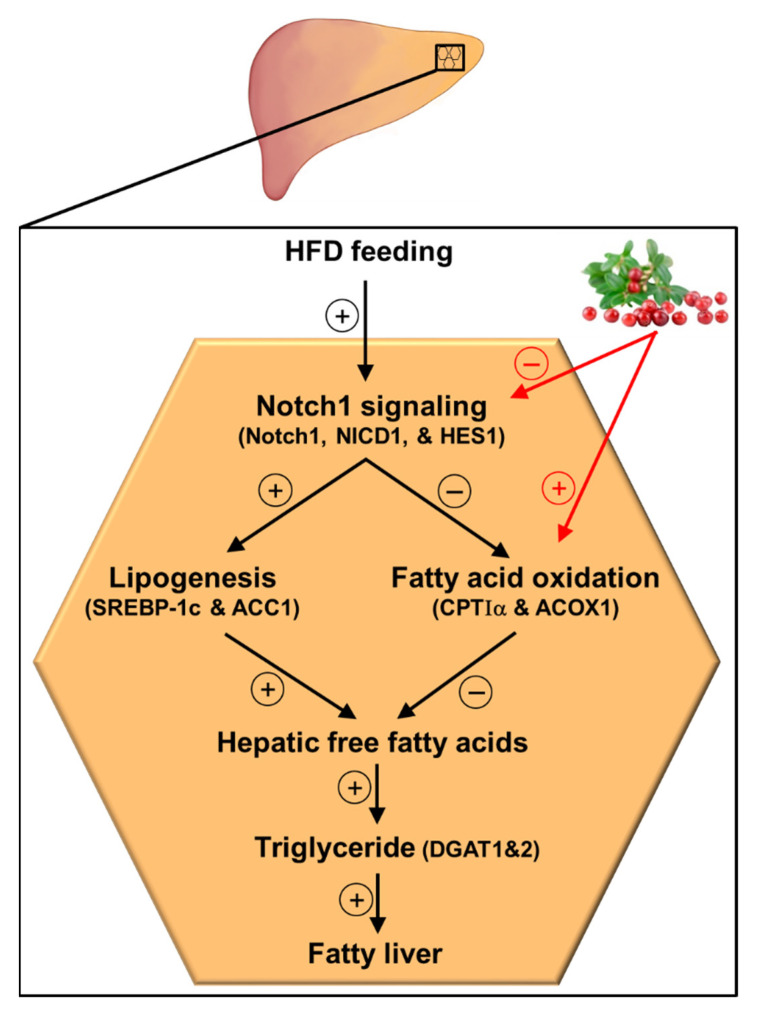
Graphical illustration and proposed mechanism for lingonberry’s effect on hepatic Notch signaling and lipid metabolism in HFD-induced NAFLD. Lingonberry protects HFD-induced fatty liver by suppressing Notch1 signaling and stimulating fatty acid oxidation. Inhibition of Notch1 signaling attenuates SREBP-1c-mediated lipogenesis and increases fatty acid oxidation identified by elevated expression of CPTIα and ACOX1. Decreased fatty acid synthesis and increased oxidation of fatty acids diminish available free fatty acids to synthesize triglycerides in the hepatocytes, depleting triglyceride stores in the liver. This will ultimately improve the fatty liver. The circled + and − symbols stand for stimulation and inhibition, respectively. Abbreviations: HFD = high-fat diet; Notch1 = Notch1 receptor; NICD1 = Notch1 intracellular domain; HES1 = hairy and enhancer of split-1; SREBP-1c = sterol regulatory element-binding transcription factor 1c; ACC1 = acetyl-CoA carboxylase 1; CPTIα = carnitine palmitoyltransferase-I-alpha; ACOX1 = acyl-CoA oxidase1; DGAT1 = diacylglycerol acyltransferase 1; DGAT2 = diacylglycerol acyltransferase 2.

In conclusion, the results of the current study demonstrate that lingonberry improved fatty liver by reducing liver lipid accumulation. Such lipid-lowering effects of lingonberry were mediated, in part, through improving hepatic lipid metabolism by inhibiting Notch1 signaling (Figure 6). Suppression of hepatic Notch1 signaling decreased SREBP-1c mediated *de novo* lipogenesis and increased the expression of key genes involved in fatty acid oxidation (Figure 6). Further, we identified that C3Glu, as one of the potential active compounds, was responsible for these beneficial effects. However, lingonberry’s lipid-lowering effect was independent of body weight change. As the global prevalence of NAFLD increases with the higher prevalence of obesity and sedentary lifestyles, alternative treatment options are required for NAFLD. Therefore, the incorporation of lingonberry into the regular diet might be an alternative option to manage NAFLD.

## Figures and Tables

**Table 1:** Primer sequences used for the RT-qPCR.

Primer	Forward Sequence (5′–3′)	Reverse Sequence (5′–3′)	Accession Number
Human			
*Notch1*	CAATGTGGATGCCGCAGTTGTG	CAGCACCTTGGCGGTCTCGTA	NM_017617.5
*HES1*	TCAACACGACACCGGATAAA	CCGCGAGCTATCTTTCTTCA	NM_005524.4
*ACOX1*	GGCGCATACATGAAGGAGACCT	AGGTGAAAGCCTTCAGTCCAGC	NM_001185039.2
*CPTI*α	CGATGTTACGACAGGTGGTTTGACA	AGTGCCCATCCTCCGCATAG	NM_001876.4
*ACC1*	TTCACTCCACCTTGTCAGCGGA	GTCAGAGAAGCAGCCCATCACT	NM_198838.2
*SREBP-1c*	ACACAGCAACCAGAAACTCAAG	AGTGTGTCCTCCACCTCAGTCT	NM_001005291.3
Mouse			
*Notch1*	CCAGCAGATGATCTTCCCGTAC	TAGACAATGGAGCCACGGATGT	NM_008714.3
*HES1*	CCCCAGCCAGTGTCAACAC	TGTGCTCAGAGGCCGTCTT	D16464.1
*DGAT1*	TTCCGCCTCTGGGCATT	AGAATCGGCCCACAATCCA	XM_006520405.4
*DGAT2*	AGTGGCAATGCTATCATCATCGT	AAGGAATAAGTGGGAACCAGATCA	NM_026384.3
*CD36*	TGTGCTAGACATTGGCAAATG	CTTCTCCTAAAGATAGGTGTG	XM_030254088.1
*ACOX1*	GCCTTTGTTGTCCCTATCCGT	CTTCAGGTAGCCATTATCCATCTCT	NM_001271898.1
*CPTIα*	CATGATTGCAAAGATCAATCGG	CTTGACATGCGGCCAGTG	NM_013495.2
*ACC1*	CGGACCTTTGAAGATTTTGTGAGG	GCTTTATTCTGCTGGGTGAACTCTC	XM_030245463.1
*SREBP-1c*	GGAGCCATGGATTGCACATT	GGCCCGGGAAGTCACTGT	NM_001358314.1

## Data Availability

Data are contained within the article.
